# Women’s employment and fertility in a global perspective (1960–2015)

**DOI:** 10.4054/demres.2020.43.25

**Published:** 2020-09-03

**Authors:** Julia Behrman, Pilar Gonalons-Pons

**Affiliations:** 1 Equal authorship, authors in alphabetical order. Northwestern University, USA.; 2 University of Pennsylvania, USA.

## Abstract

**BACKGROUND:**

Scant research explores the association between women’s employment and fertility on a truly global scale due to limited cross-national comparative standardized information across contexts.

**METHODS:**

This paper compiles a unique dataset that combines nationally representative country-level data on women’s wage employment from the International Labor Organization with fertility and reproductive health measures from the United Nations and additional information from UNESCO, OECD, and the World Bank. This dataset is used to explore the linear association between women’s employment and fertility/reproductive health around the world between 1960 and 2015.

**RESULTS:**

Women’s wage employment is negatively correlated with total fertility rates and unmet need for family planning and positively correlated with modern contraceptive use in every major world region. Nonetheless, evidence suggests that these findings hold for nonagricultural employment only.

**CONTRIBUTION:**

Our analysis documents the linear association between women’s employment and fertility on a global scale and widens the discussion to include reproductive health outcomes as well. Better understanding of these empirical associations on a global scale is important for understanding the mechanisms behind global fertility change.

## 1. Introduction

There have been dramatic global transformations in women’s status around the world in recent history. One particularly striking transformation has been global changes in women’s labor force participation, which has increased around the world over the last century (ILO 2018a).^3^ Globally, women make up about 40% of the world’s workforce, including an increasing number of women in low- and middle-income countries, especially in agriculture, manufacturing, and service sectors (ILO 2015). Over a similar time period, there have also been important changes in global fertility patterns, including falls in total fertility rates (TFRs) in most major regions of the world (de Silva and Tenreyro Forthcoming; Dorius 2008; Morgan 2003; Wilson 2001). Estimates suggest that global TFR fell from about 5 in 1960 to just under 2.5 in 2015, representing a staggering transformation in global fertility trends (de Silva and Tenreyro Forthcoming).

Given that both employment and fertility are intimately tied to women’s economic and social statuses in families and societies, there has been enormous interest in the correlation between women’s employment and fertility. In high-income countries, the negative correlation between women’s wage employment and fertility has been well documented (Ahn and Mira 2002; Bernhardt 1993; Brewster and Rindfuss 2000; Moen 1991; Waite 1980), although there has been some evidence of a reversal in these trends in some contexts in recent decades due to adoption of policies that reconcile employment and family conflict (Brewster and Rindfuss 2000; Rindfuss and Brewster 1996). There has been less research overall on the employment–fertility correlation in low- and middle-income countries than in high-income countries, perhaps due to the enormous heterogeneity in prevalence and type of employment across these contexts. In one notable exception, Bongaarts and colleagues document a negative association between having children at home and women’s employment in low- and middle-income countries, albeit with heterogeneity by region and type of employment (Bongaarts, Blanc, and McCarthy 2019). For example, employment in agriculture has close to a null relationship with having children at home, but employment in transitional sectors (e.g., household/domestic service) or modern sectors (e.g., professional, managerial, clerical) is negatively associated with the number of children at home.

To the best of our knowledge, there is limited to no work that explores the correlation between women’s employment and fertility on a truly global scale. In part, this lack of global exploration on the topic is due to data constraints, since it is difficult to find cross-national comparative standardized information about employment, fertility, and reproductive health in survey data across high- and low-income contexts. For example, standardized IPUMS census micro-data contain information about current employment and children residing in the household but not total fertility or reproductive health outcomes. Other commonly used cross-national data sources – such as the Luxemburg Income Study or Demographic and Health Surveys – are only available for a subset of countries that are typically at similar levels of socioeconomic development. Furthermore, because measures vary substantially across surveys, it is challenging to find standardized measures of women’s employment, including both salaried employment and informal piecemeal employment, the latter of which is particularly common in low- and middle-income countries (ILO 2018b).

This paper compiles a unique global dataset that combines nationally representative data on women’s wage employment from the International Labor Organization (ILO) with fertility measures from the United Nations (UN) and additional information from UNESCO, OECD, and the World Bank. All our analyses are conducted at the country level and thus explore aggregated – and not micro-level – associations between employment and fertility/reproductive health. The advantage of using aggregated data is that the experience of living in a country where many women are employed may have important spillover effects even among unemployed women, and these may be captured in our analyses. For example, high levels of women’s employment in a society may correspond with broader sociocultural shifts in norms about gender, fertility, and fertility regulation even among women who are not employed but who are exposed to new role models, norms, and ideas by seeing other women in the public sphere.

In what follows we highlight dominant approaches that have been used to understand the associations between women’s employment and fertility/reproductive health in literature from high- and low-income countries. Although these explanations sometimes focus on a unidirectional relationship (e.g., the effects of fertility on employment or the effects of employment on fertility), we emphasize that this relationship could run in either direction (or both). Next we explore the linear associations between women’s wage employment and TFR at the country-level from 1960 onward for four major world regions, encompassing both high-, middle-, and low-income countries. Because women’s abilities to regulate their fertility via modern contraceptive methods could be an important cause and consequence of their entrance into the labor force, we also explore the linear associations between women’s modern contraceptive use and unmet need for family planning. In doing so, our analysis widens the discussion of the fertility and employment correlation to include reproductive health outcomes beyond fertility. Finally, we explore the linear associations between employment and TFR, contraceptive use, and unmet need for family planning, disaggregating by whether or not the employment is in the agriculture sector, thus providing insight into whether the type of employment matters for these linear associations. Although we are not able to estimate causal impacts in this paper, descriptive associations are nonetheless important for furthering understandings of the relationship between employment and fertility across diverse global settings.

## 2. Approaches to the employment–fertility correlation

### 2.1 The incompatibility approach

The dramatic expansion of women’s labor force participation in high-income countries in the last century represented a major change in women’s status within families and societies and corresponded with important shifts in fertility and family formation (Goldin 1995, 2006). A fairly extensive body of literature has examined the premise that the incompatibility between employment and child-rearing leads to reductions in fertility (Brinton and Lee 2016; McDonald 2000b, 2006), reductions that in some cases have led to the lowest fertility levels documented in several European contexts (Esping-Andersen and Billari 2015; Kohler, Billari, and Ortega 2002). Although this approach sometimes assumes that that employment will affect fertility decision-making, women’s abilities to regulate and lower their fertility are also important precursors to their employment (Aguero and Marks 2008; Angrist and Evans,1998; Bailey 2006; Bloom et al. 2009; Cáceres-Delpiano 2012; Cruces and Galiani 2007; Rosenzweig and Wolpin 1980). For example, it has been shown that the introduction of hormonal birth control was important for expanding women’s labor force participation in the United States (Bailey 2006; Goldin and Katz 2002).

The incompatibility hypothesis hinges on the nature of employment in industrialized economies. The idea is that in industrialized economies, unlike other economies, employment and moneymaking activities are more incompatible with child-rearing because they take place outside the house and under a time schedule that is more inflexible than employment performed in the house (Stycos and Weller 1967; Weller 1977). The implication is that women’s employment is compatible with high fertility in preindustrial agricultural settings but less so in industrialized economies. At the individual level, research in high-income countries shows that women who are employed have fewer children that women who are not employed (Spain and Bianchi 1997). Furthermore, pursuing a career tends to delay the onset of fertility for logistical or social reasons, which ultimately lowers completed fertility (Rindfuss and Brewster 1996).

At the aggregate level, the incompatibility hypothesis suggests that there should be lower levels of fertility in countries with higher levels of women’s employment. Studies show, however, that the translation of the individual-level mechanism to the aggregate level is not always straightforward. Research in high-income countries shows that high levels of women’s employment have been correlated with lower fertility in the past, but in recent decades there has been a positive association between levels of women’s employment and fertility in some contexts (Brewster and Rindfuss 2000; Rindfuss and Brewster 1996). The main explanation developed to account for this reversal and the compatibility/coexistence of very high levels of employment and relatively “high” fertility has focused on social policy and institutions, and changes in gender relations. On the one hand, countries might set up institutions that reduce some of the incompatibilities between employment and child-rearing (e.g., parental leave, child-care centers, part-time and flexible employment) (Esping-Andersen and Billari 2015; Goldscheider, Bernhardt, and Lappegård 2015). At the same time, changes in gender relations that result in men’s increased involvement in child-rearing might similarly reduce the negative association between employment and fertility. Nonetheless, the relationship between institutions and changes in gender relations is partly endogenous, as certain forms of social policy can trigger changes in gender relations and shifts in gender relations can increase demand for institutional change.

Of course, there is considerable complexity in the social meanings of employment, and these may change over time as women’s economic opportunities are transformed by changing social and economic circumstances. For example, as more and more women join the labor force, increasing numbers of women may come to see employment as a viable possibility, thus leading to higher opportunity costs for childbearing and lower preferences for fertility (Becker and Lewis 1974). At the same time, increases in women’s labor force participation at the national level may change women’s perceptions about the possibility or acceptability of working while a child is young (particularly if there are family policies that help facilitate work–family incompatibilities), which could actually lead women to perceive lower opportunity costs and higher childbearing desires. Whether or not increases in women’s labor force participation lead women to perceive higher or lower opportunity costs to childbearing may be heterogenous across contexts and may depend on the starting level of women’s employment in society. Furthermore, this may change over time as policies and norms also change.

Although the incompatibility approach is typically applied to industrialized settings where women are employed outside the home, it could also be useful in low-income preindustrial settings where women must simultaneously balance many different types of paid and unpaid labor. For example, a randomized control trial in informal settlements in Nairobi, Kenya, found that subsidized child care led to significant increases in poor urban women’s employment (Clark et al. 2019). This finding runs counter to the assumption that women’s child-care responsibilities are not obstacles to their employment in low-income preindustrial settings, where women are assumed to have more flexibility and nearby family to help. This suggests that incompatibility may be a more important part of the fertility–employment explanation than is often considered in low-income settings where women engage in paid employment in both formal and informal situations.

### 2.2 The empowerment approach

Another approach suggests that earned income is an important determinant of women’s autonomy; thus women’s employment is an important form of economic empowerment that is important for fertility reduction (Upadhyay et al. 2014; Upadhyay and Hindin 2005). Although there has been debate on what exactly empowerment entails (Kabeer 1999), it has been a widely utilized concept in research on low-income contexts. The idea underlying this approach is that women’s employment can lead to a radical transformation in their options for economic survival and their bargaining power within families, including the ability to advocate for their own fertility desires (Anderson and Eswaran 2009; Duflo 2012; Narayan-Parker 2005). Just as the opening of jobs for young men lowers fathers’ patriarchal power over them (Ruggles 2015), women’s employment reduces their dependency on family ties (including fathers as well as husbands) by providing them with independent sources of income.

In contexts where women’s lack of choice over their reproduction is part of a broader patriarchal regime, where women often also lack access to reproductive health care, contraceptives, and abortion (Barber et al. 2018), women’s increased financial resources could give them more bargaining power to advocate for their reproductive preferences (Allendorf 2007; Beegle, Frankenberg, and Thomas 2001; Behrman 2017; Doss 2005; Quisumbing and Maluccio 2003). In further support of this, there is evidence linking women’s economic autonomy (measured as access to paid employment or micro-credit loans) to higher family planning use in South Asia (Dharmalingam and Morgan 2004; Schuler, Hashemi, and Riley 1997). At the same time, the reverse may be true as well, as increased access to reproductive control and lowered fertility may empower women in new dimensions, including by allowing them to enter the wage labor market.

Nonetheless, women’s employment is not always empowering, particularly given the considerable heterogeneity in types of employment women perform across contexts. Many women around the world are employed in the informal economy in jobs that lack security or stability and are physically and mentally strenuous (ILO 2018b). Many women are also disadvantaged in maintaining control over employment-related resources and earnings (Ferber, Green, and Spaeth 1986). Throughout low- and middle-income countries, the proportion of women engaged in informal employment is higher than the proportion of men, which has implications for women’s abilities to obtain and negotiate for decent income and safe labor conditions.^4^ In many regions – including South Asia, the Middle East, and North Africa – a considerably higher proportion of women’s employment than men’s employment is concentrated in agriculture (ILO 2015) because men have left agriculture to pursue better opportunities in service and manufacturing sectors. Informal and/or poorly paid jobs (which are in many regions concentrated in agriculture) may be less effective at changing women’s preferences or bargaining abilities because the women holding these jobs lack financial security and/or personal autonomy.

It is also plausible that only jobs that take women outside the direct patriarchal authority of male relatives are effective at increasing women’s autonomy. For example, Anderson and Eswaran (2009) find that employment does not inherently lead to increased women’s autonomy in Bangladesh. Rather, employment needs to be outside of husbands’ farms to positively affect female autonomy outcomes. This is relevant because around the world, a disproportionate share of women also can be considered “contributing family workers” (e.g., employed in a market-oriented enterprise owned by a household member) (ILO 2016). This is particularly the case in sub-Saharan Africa and southern Asia, where the percentage of women who are contributing family workers exceeds that of men by 18 percentage points and 23 percentage points, respectively (ILO 2016).

Although the empowerment approach has primarily been applied to low-income countries where many women are entering the labor market for the first time, there are aspects of the empowerment perspective that could be useful for high-income countries as well. Policy makers often assume that incompatibility between child-rearing and employment is the main cause of low fertility in high-income settings. While policies that promote work–family balance can indeed have important social benefits, the introduction of generous family policy is not a panacea for low levels of fertility (Chesnais 1996; Hoem 1990; McDonald 2006). This could reflect that men’s care burden has been slow to change in many contexts, but it could also speak to the fact that the wide-scale entrance of women into the labor market has led to broader changes in values and norms about desired childbearing. Women might want fewer children (at least partially) not just because of incompatibility but because they find social meaning in other aspects of life outside of motherhood and have the resources to realize their goals (Blackstone and Stewart 2012).

## 3. Data, measures, and methods

### 3.1 Data

We draw on multiple sources to construct a unique global time-series dataset on women’s employment, fertility, and reproductive health trends for low-, middle-, and high-income countries. All measures and analyses are conducted at the country level, and we strive to include as many country-years as possible. Data on employment are taken from the International Labor Organization; data on fertility and reproductive health are taken from Global UN; and data on economic and schooling conditions are taking from UNESCO, OECD, and the World Bank (via the World Bank data archive). Our current sample focuses on adult populations and includes 174 countries ranging across the years 1960–2015, representing 89% of the 195 countries in the world. Table 1 presents a summary of key measures by region. Our dataset has information on most of the largest countries in the world (including China, India, the United States, and Brazil). We present estimates for the pooled global sample and also aggregate countries into four major regions: (a) Europe, United States, Canada, Australia, and New Zealand (which for simplicity we refer to as Europe/North America), (b) Latin America, (c) Africa, and (d) Asia. The regions are grouped using a modified version of the UNSD M49 region code, although for reasons of linguistic and sociocultural similarity we include Australia and New Zealand with the United States and Europe rather than Asia. Appendix Table A-1 lists countries included in each region.

### 3.2 Measures

#### Women’s employment

Women’s employment is a central measure in our analysis because it has long been hypothesized to be both a cause and a consequence of fertility change. We measure women’s employment using ILO data on the employment-to-population ratio for women, which is calculated by dividing the number of women employed by the number of women in the working-age population (i.e., aged 15–65) and multiplying by 100. The ILO defines the employed as “all persons of working age who during a specified brief period, such as one day or one week, were in the following categories (a) paid employment (whether at work or with job but not at work); or (b) self-employment (whether at work or with an enterprise but not at work)” (ILO 2019). Typically, the working-age population is 15 to 65, although there is some country-level variation in what is considered working age. A high ratio of employment to population means that a large share of the population of working-age women is employed, whereas a low ratio of employment to population means that a large share of the population of working-age women is either unemployed or out of the labor market. ILO estimates are based on country labor force surveys. For detailed information on ILO’s standardization process. see Bourmpoula, Kapsos, and Pasteels (2016).

Employment is highly heterogenous (i.e., there are differences in skill sets, compensation, levels of formality, and so on), so we also explore whether the type of employment matters for the employment–fertility correlation. Because available literature suggests that the central fissure is between agricultural and nonagricultural employment (particularly in low- and middle-income countries) (Bongaarts, Blanc, and McCarthy 2019), we also conduct analyses with alternative employment measures (also taken from the ILO) that capture women’s employment in agricultural versus nonagricultural activities. The measures are the share of women employed in agriculture over all women employed, and the share of women in nonagriculture over all women employed. Linear interpolation is used for country-years with missing values in both employment variables.^5^ Because not all countries have agricultural employment data, as a robustness check, we rerun all our main models, restricting the sample to the countries that do have agricultural data; results are substantively the same and are available upon request.

#### Fertility

Fertility is hypothesized to be important because employment might lead women to lower their childbearing (due to incompatibility, empowerment, or some combination of both) or because lowered childbearing allows women to seek employment. In our analysis, fertility is measured as the TFR in any given year. The TFR is a synthetic measure of fertility that approximates the number of children a woman would have if she were to experience age-specific fertility levels in a given year. It is important to note that TFR is age standardized (other measures used in this analysis are not). TFR data come from UN Population (2017). The UN calculates the TFR using data from civil registration systems, household surveys, and censuses.^6^ Linear interpolation is used for country-years with missing values of this variable using the same strategy as described above.

#### Modern contraceptive use

Modern contraceptive use is an important proximate determinant of fertility: Increased usage of modern contraception might allow women to seek employment. Alternatively, employment might lead women to adopt modern contraceptive measures by providing them with the financial autonomy necessary to access contraceptives or the motivation to regulate conception. Modern contraceptive use could be an active choice of women who want to regulate fertility, but women may also use modern contraceptives with limited volition at the instruction of partners, medical professionals, or NGO workers. Modern contraceptive use is measured as the proportion of women of reproductive age (15–49) who report current use of any modern contraceptive methods, including oral contraceptive pills, implants, injectables, intrauterine devices, male condoms, female condoms, male sterilization, female sterilization, lactational amenorrhea, and emergency contraception. These estimates are taken from UN Population and are calculated using nationally representative survey data (Kantorova 2019).

#### Unmet need for family planning

Unmet need for family planning is an important measure of whether women want to stop or limit childbearing but are not using modern methods, presumably due to factors such as lack of access or knowledge. This is relevant because employment might lead to lower unmet need for family planning if employment corresponds with women’s autonomy and control over resources. At the same time, low unmet need for family planning might also lead to higher women’s employment because women are confident they can regulate fertility in ways that allow them to pursue paid employment without interruption. Although unmet need for family planning is related to modern contraceptive use, it is conceptually distinct because it captures unrealized needs, whereas contraceptive use captures actual usage (although usage might be determined by oneself or another person). Unmet need is measured in accordance with international standards as the proportion of women of reproductive age (15–49) who want to stop or delay childbearing but are not using a modern method of contraception.^7^ These estimates are taken from UN Population and are calculated using nationally representative household survey data (Kantorova 2019).

#### Gross domestic product

Gross domestic product (GDP) is important because underlying economic conditions are likely correlated with both women’s employment opportunities and their fertility outcomes. GDP could also be causally intermediate, because expanded women’s work might impact GDP, which in turn might impact fertility. GDP is a time-varying country-level measure of economic conditions that is calculated in current US dollars and is retrieved from the World Bank based on calculations using World Bank national accounts data and OECD national accounts data.

#### Schooling

Schooling is positively correlated with both women’s labor force participation and negatively correlated with women’s fertility. Schooling is measured by the school enrollment secondary (gross) gender parity index (GPI). GPI is calculated as the ratio of girls to boys enrolled at the secondary level in public and private schools. A GPI of less than 1 suggests that girls have a disadvantage in secondary education, and a GPI of greater than 1 suggests that girls have an advantage in secondary education. GPI is retrieved from the World Bank and based on data from the UNESCO Institute for Statistics. As a robustness check, we rerun all models, substituting GPI with a measure of the percent of women who completed secondary education; this measure is retrieved from the World Bank using data from UNESCO. We do not include secondary education in our main models because we lose about 800 observations from 20 countries due to missing data on this measure (although all general patterns are robust to including this measure).

### 3.3 Methods

We start by graphing country-level trends in employment and TFR to provide a descriptive overview of how employment and fertility are changing globally. As a next step, we assess the linear associations between country-level women’s employment and TFRs (including country fixed effects). Because the relationship between employment and fertility is likely bidirectional – employment might influence fertility, but fertility could also influence employment – our estimates capture a linear association but with no assumptions about directionality. (In other words, we make no assumptions about whether women’s employment affects fertility or vice versa.^8^) We run these models for a pooled global sample of all countries in our analysis and disaggregated by the four regions. While the estimates we use are representative at the country level (using country weights when appropriate), because country-years are the main units of the main analysis, we do not weight by country size when pooling countries in the regional and global analyses. Instead, we treat each country equally, which ensures that changes in employment/fertility in large countries do not disproportionately affect our pooled estimates. This strategy has been employed by others conducting similar analyses (Pesando et al. 2019).

Changes in both women’s employment and fertility likely correspond with myriad other social and economic changes. Thus, as a supplement, we also run a second set of models where we include controls for time-varying country-level factors such as GDP and GPI. Because there are many unobserved time-varying factors not included in our models (e.g., population age structures, governmental or policy changes, patterns of internal or external migration), it is important to emphasize that these analyses capture associations and not causal effects.

The literature suggests that the type of employment is consequential for fertility outcomes and that only certain types of employment (such as nonagricultural, salaried, and outside the family) might be correlated with women’s financial autonomy and/or fertility and reproductive health outcomes (Anderson and Eswaran 2009; Finlay 2019). Given this, we also run models where we disaggregate the correlations by agricultural versus nonagricultural employment.

Because women’s ability to regulate their fertility via modern contraceptive methods could be an important cause and consequence of entrance into the labor force, we also explore the linear associations between women’s unmet need for family planning and modern contraceptive use, using the same empirical strategy. This provides a fuller analysis of the association between women’s employment and reproductive health beyond just fertility.

While the age ranges for the variables of interest differ (employment measures are calculated for the working-age population of 15 to 65, and contraception measures are calculated for the reproductive age population of 15 to 49), we do not necessarily see this as a limitation, since we use aggregated measures of these variables. For example, it is plausible that women in the reproductive years may be influenced by large numbers of older women who are still employed. By including country fixed effects, we make sure that the estimates are an average of within-country variation in associations between employment and fertility/reproductive health, but these estimates do not draw on between-country differences in other characteristics, such as population age structure.

## 4. Results

### 4.1 Descriptive results: Women’s employment and fertility in a global perspective

**Figure 1:**
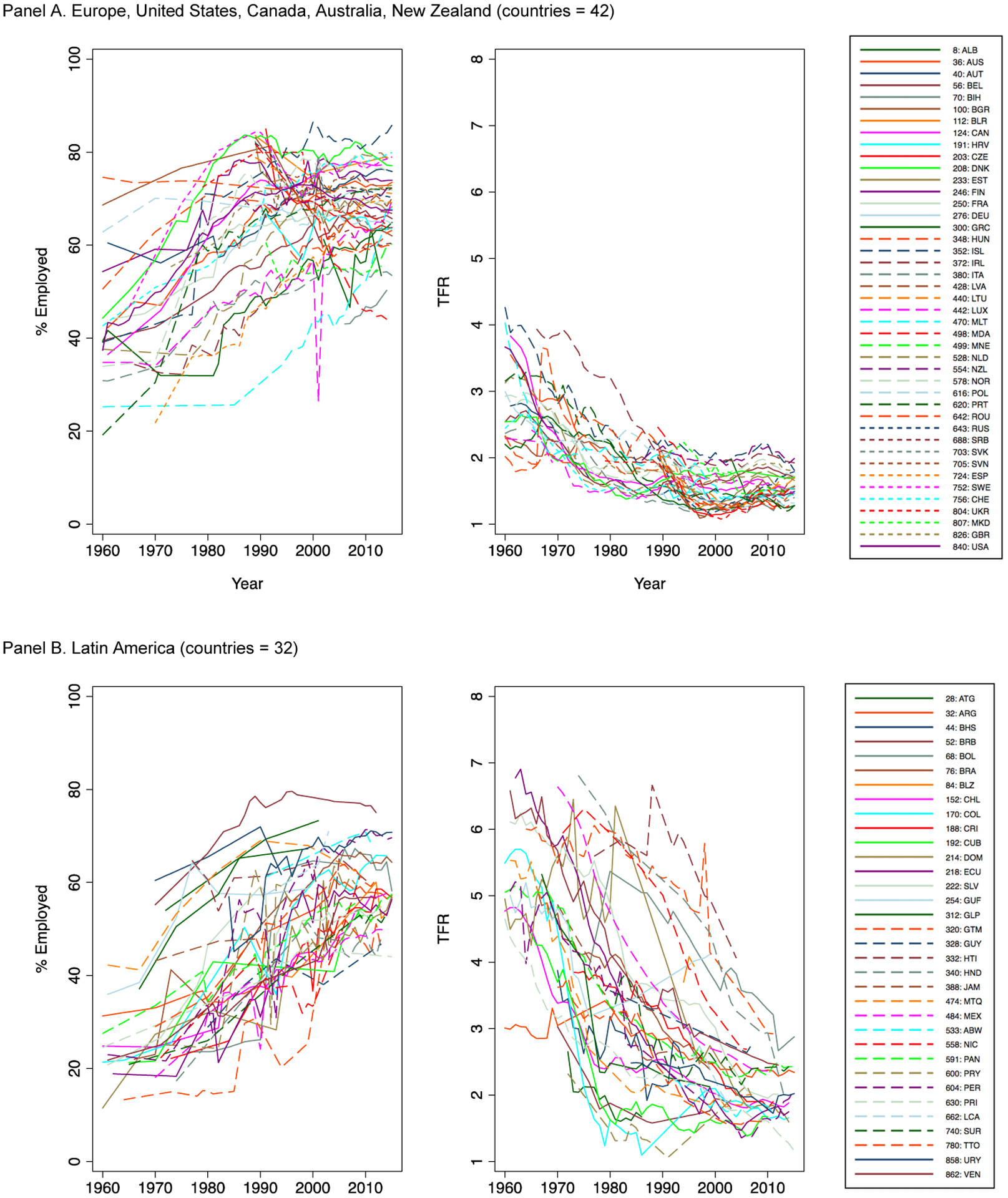

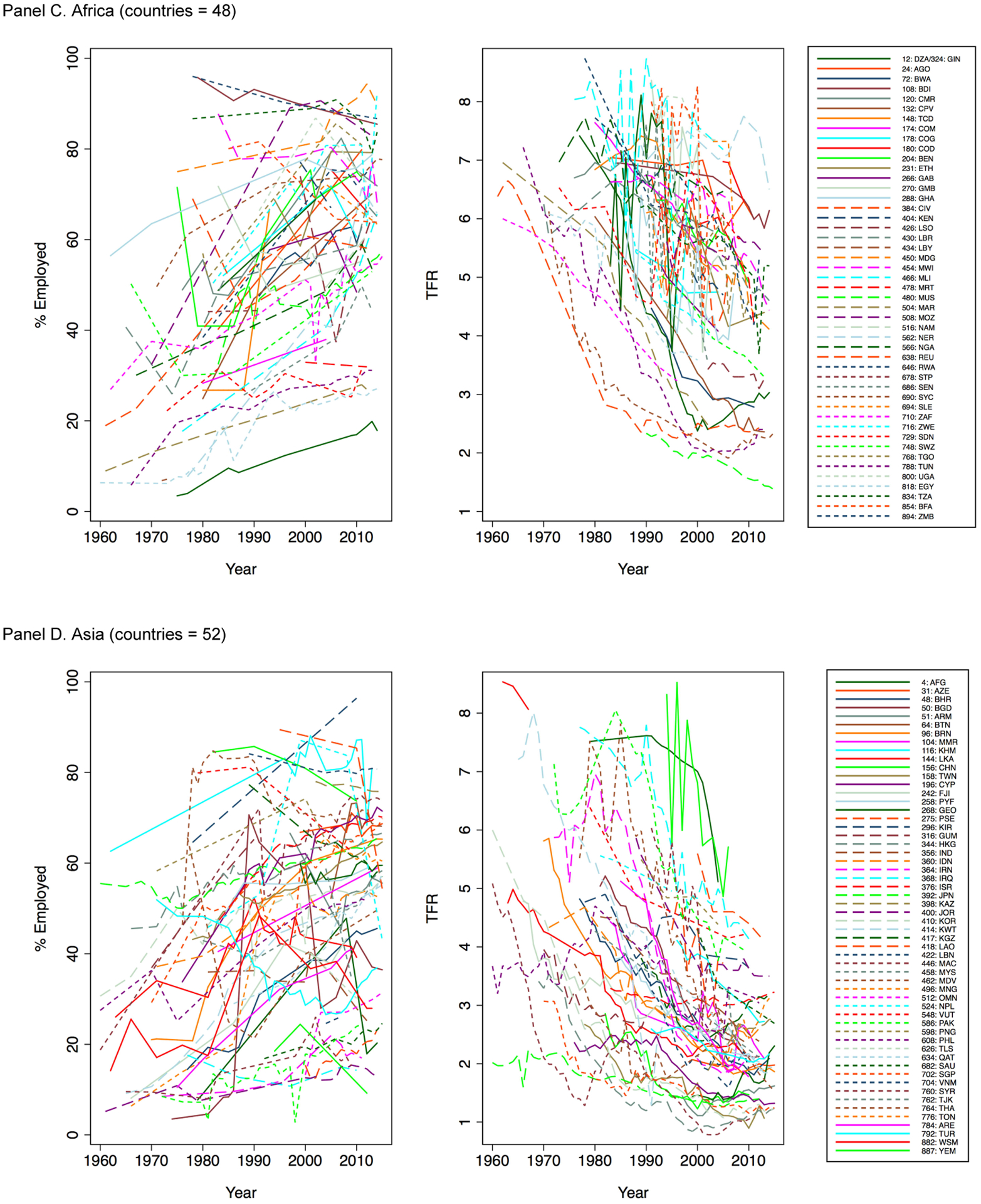
Global employment and fertility trends, 1960–2015 *Source*: Created by the authors using data from ILO and UN.

Figure 1 shows women’s employment and total fertility rates for all country-years by geographic region. Despite variation in levels and trends, these descriptive results overall suggest both increasing women’s employment and declining fertility across regions. Panels A and B (Europe/North America and Latin America) show this pattern most clearly, while Panels C and D (Africa and Asia) display more heterogeneity.

Panel A (Europe/North America) shows the well-known increase in women’s employment, which begins as early as the pre-1960s for some countries and as late as the 1980s for others. These changes in employment coincide with moderate but meaningful declines in fertility, as fertility levels drop well below replacement levels. Our data also show a timid rebound in total fertility in the 2000s, which other researchers have used to suggest that shifts in policies and gender norms can work to mitigate the incompatibility between employment and fertility (Goldscheider, Bernhardt, and Lappegård 2015). Panel B, on Latin America, also shows striking increases in women’s employment and declines in fertility levels. Unlike Panel A, however, declines in fertility begin from much higher levels and do not generally drop below replacement levels in most places. The overall increase in women’s employment in this period is comparable to that experienced in high-income countries (Panel A), although the overall levels are generally lower.

Panels C and D show trends in Africa and Asia. Employment levels and trends are highly heterogeneous in both regions. In Africa, women’s employment rates are generally flat. Some countries have high employment rates (such as Malawi and Kenya, at 70%), while others have very low employment rates (such as Egypt and Algeria, at about 10%–25%). The enormous heterogeneity in Africa likely reflects that many employment opportunities in Africa are informal and piecemeal in nature (e.g., agricultural labor and selling in markets) (Al Samarrai and Bennell 2007; Hino and Ranis 2014). In Asia, employment rates are similarly varied, which also likely reflects the high level of informal and often precarious labor. Nonetheless, there are small increases over time in women’s employment, which could reflect rises in female-oriented service and manufacturing jobs and also rising urbanization. Fertility trends in Africa and Asia are also heterogeneous. Most countries show moderate declines, although fertility levels vary greatly. For instance, in Cape Verde, the total fertility rate drops from 6.2 to 2.3 between 1978 and 2013, whereas in Cameroon, drops were more moderate (e.g., from 6.6 to 5.7) over a similar period. Nonetheless, the overall high levels of fertility and the great heterogeneity in levels of women’s employment mean the correlation between women’s employment and fertility is less clear in these two regions.

### 4.2 Linear associations between women’s employment and TFR

**Figure 2:**
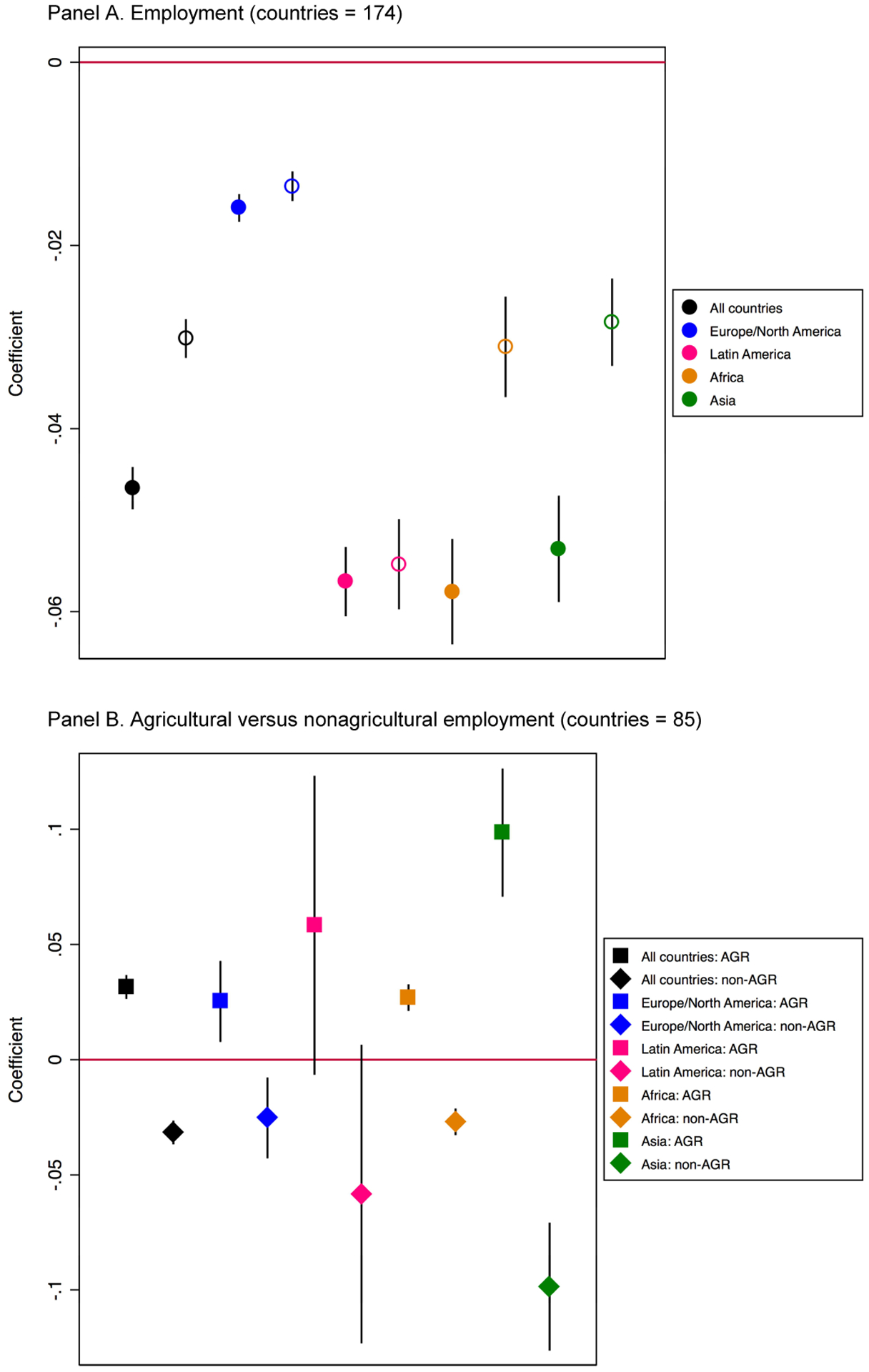
Linear association between wage employment and TFR with country fixed effects (1960–2015). Panel A shows the empty model (solid dots) and the model with controls for GDP and GPI (hollow dots). Panel B disaggregates by agricultural versus nonagricultural employment. *Source*: Created by the authors using data from ILO, UN, and World Bank.

The preceding section showed descriptive evidence that women’s employment increased, and fertility decreased, in all four major world regions, albeit with within-region heterogeneity. In Figure 2, Panel A reports results from regressions that test for a statistically significant linear association between women’s wage employment and TFR at the country level. Our main model, Model 1, adjusts only for country fixed effects and is represented by the solid dot. Model 2 includes controls for GDP and GPI and is represented by the hollow dot. We run Models 1 and 2 for the pooled sample of all countries and for each of the four regions in our analysis. We present results as a series of figures; corresponding regression tables can be found in Appendix Tables A-2 to A-7.

In the pooled estimates – represented by the black dot – there is a statistically significant negative association between women’s employment and TFR in both Model 1 and Model 2. When we disaggregate by region, we see there is a negative association between employment and TFR in all four regions. Nonetheless, the magnitude of the employment–fertility correlation is considerably smaller in Europe/North America – represented by the solid blue dot – than in the other three world regions, which may reflect more work–family reconciliation policies in this region. The larger confidence intervals on the point estimates for Latin America (pink), Africa (orange), and Asia (green) compared to Europe/North America likely reflect the larger heterogeneity in levels of women’s employment and TFRs across contexts in these regions. Including controls for GPI and GDP in Model 2 does little to alter the magnitude or the significance of coefficients for Europe/North America or Latin America. In Africa and Asia, the magnitude of the employment–fertility correlation becomes smaller upon adding these controls (though it retains statistical significance).

In Figure 2, Panel B presents results of the linear association between women’s employment and TFR, disaggregating by agricultural employment versus nonagricultural employment. In the pooled model of all regions, women’s agricultural employment is positively associated with TFR (black square), but women’s nonagricultural employment is negatively associated with TFR (black diamond). The general pattern of a positive correlation between agricultural employment and TFR and a negative correlation between nonagricultural employment and TFR is echoed in the region-specific analyses, although not all of these coefficients are statistically significant at *p* < 0.05. This may be due to reduced sample sizes for the agricultural/nonagricultural employment analysis, which falls from 174 countries to 85 countries in the pooled analysis due to less data about type of employment being available in many countries. This may limit statistical power, particularly in the region-specific analyses, where samples fall even further.

### 4.3 Linear associations between women’s employment, contraceptive use, and unmet need for family planning

**Figure 3:**
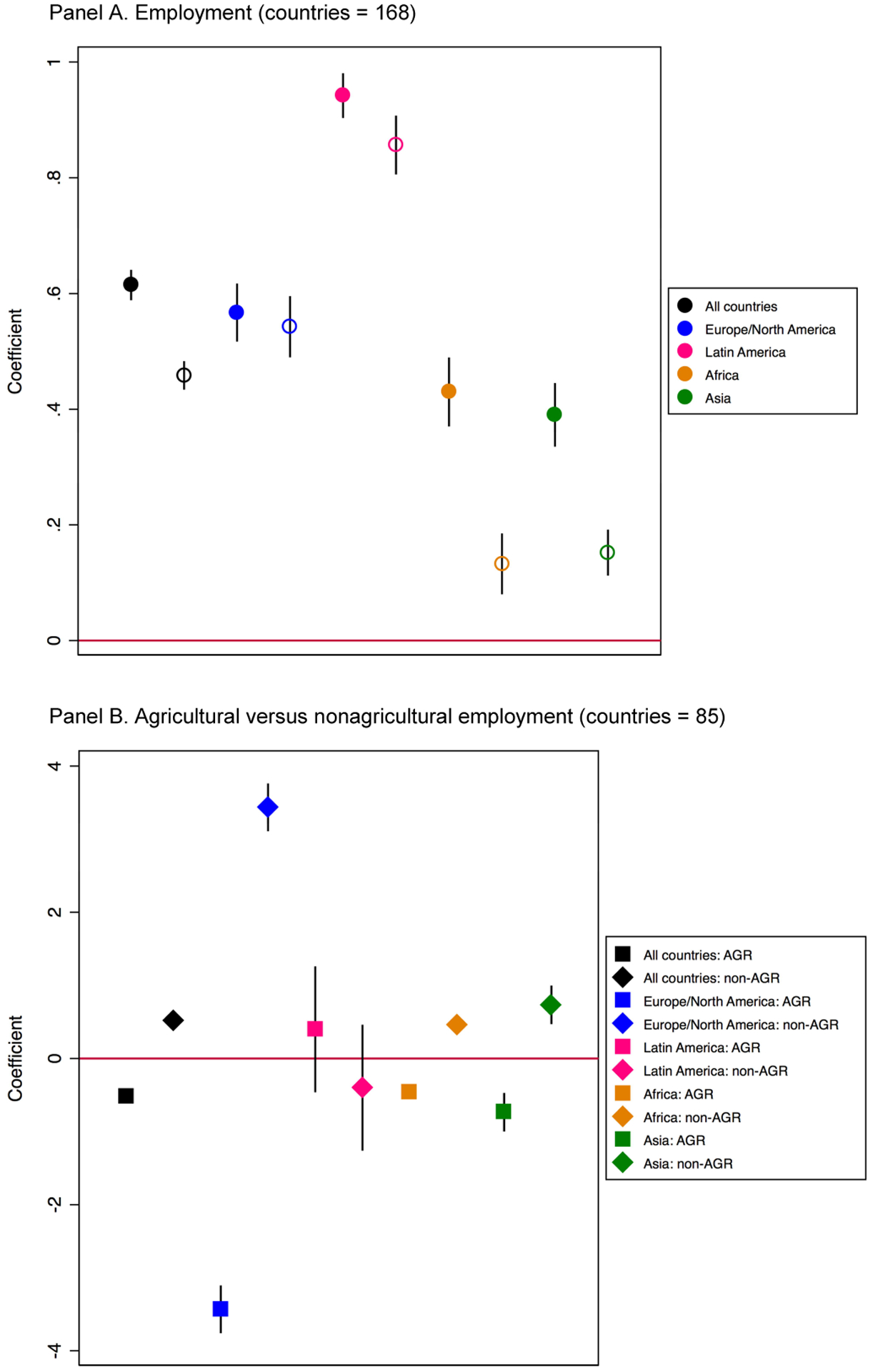
Linear association between wage employment and modern contraceptive use with country fixed effects (1960–2015). Panel A shows the empty model (solid dots) and the model with controls for GDP and GPI (hollow dots). Panel B disaggregates by agricultural versus nonagricultural employment. *Source*: Created by the authors using data from ILO, United Nations, and World Bank.

Our next set of models uses the same empirical strategies to explore linear associations between women’s employment and fertility regulation via contraceptive use. As Figure 3, Panel A, shows, there is a significant positive association between women’s employment and modern contraceptive use in both the pooled sample and in all four regional analyses (this is true with and without controls). Nonetheless there is important regional heterogeneity in the magnitude of the coefficients: The association between women’s employment and modern contraceptive use is significantly higher in Latin America (pink dot) and lower in Africa and Asia (orange and green dots), net of controls for GDP and GPI. Similar to what we documented with TFR, the relationship of interest varies by type of employment. Figure 3, Panel B, shows that women’s agricultural employment is negatively associated with modern contraceptive use (black square) and that women’s nonagricultural employment is positively associated with modern contraceptive use (black diamond) in the pooled model. This general pattern holds in the region-specific analyses as well, although some of the coefficients fail to reach statistical significance at *p* < 0.05, likely due to reduced sample size, which falls from 168 countries to 85 in the pooled analysis due to lack of data on type of employment.

**Figure 4:**
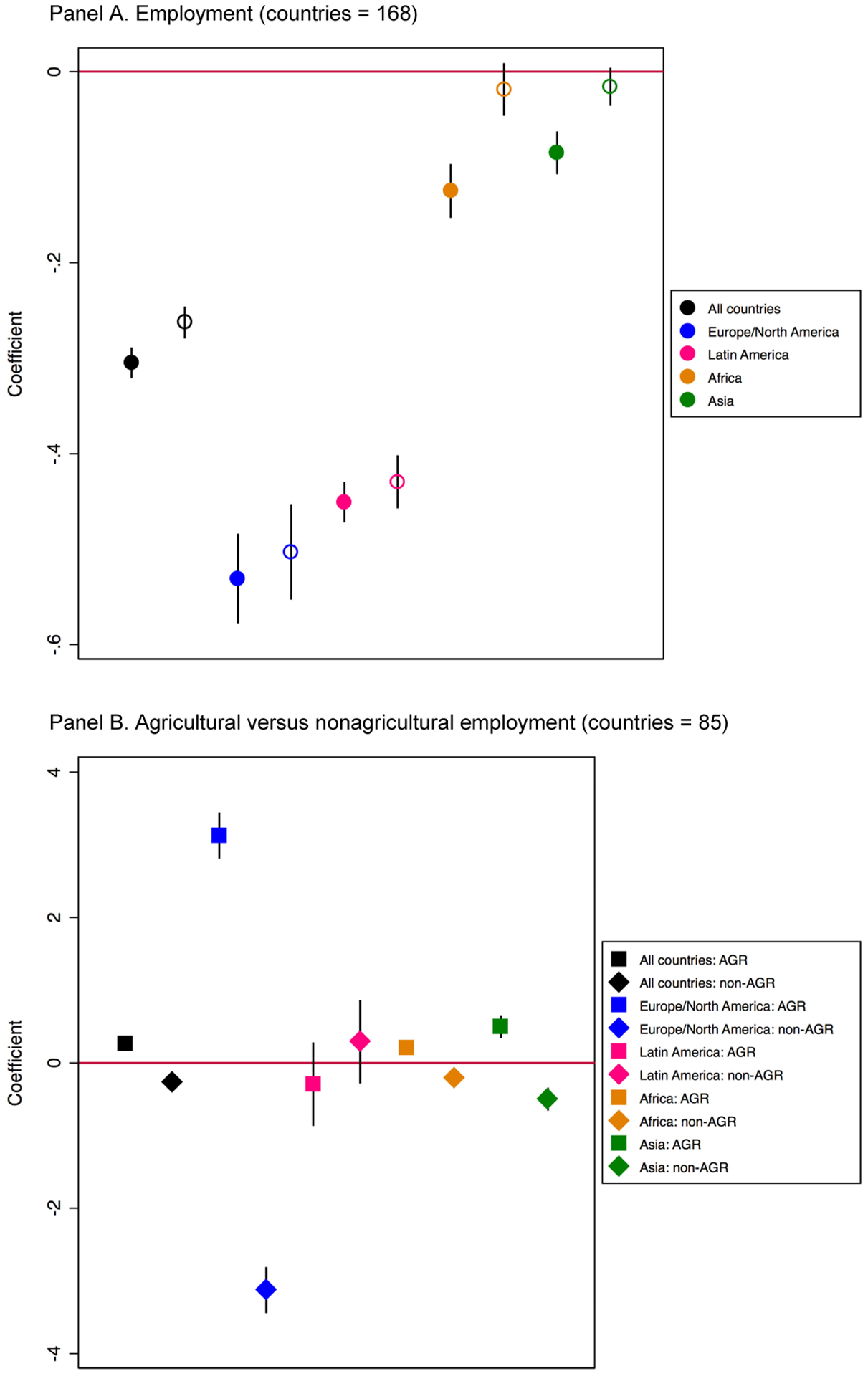
Linear association between wage employment and unmet need for modern methods of family planning with country fixed effects (1960–2015). Panel A shows the empty model (solid dots) and the model with controls for GDP and GPI (hollow dots). Panel B disaggregates by agricultural versus nonagricultural employment. *Source*: Created by the authors using data from ILO, United Nations, and World Bank.

Figure 4, Panel A, presents results of the linear association between women’s wage employment and unmet need for family planning, documenting a significant negative association between women’s employment and unmet need for family planning in both the pooled sample and all four regions (although the Africa and Asia coefficients fail to achieve significance at *p* < 0.05 upon including controls for GDP and GPI). Also of note is that the magnitude of the employment–unmet need correlation is significantly larger in Latin American (pink dot) and Europe/North America (blue dot) than in the other regions. Once we disaggregate by type of employment in Figure 4, Panel B, we see that agricultural employment is positively associated with unmet need for family planning and that nonagricultural employment is negatively associated with unmet need for family planning in the pooled analysis, a pattern that holds in the region-specific analyses as well, although some of the coefficients fail to reach statistical significance at *p* < 0.05, likely due to reduced sample size in this sub-analysis.

## 5. Discussion

This paper expands the scope of the literature on women’s employment and fertility to a truly global scale by compiling a unique dataset on women’s wage employment and reproductive outcomes in low-, middle-, and high-income countries. Our analyses document a significant negative linear association between women’s wage employment and the total fertility rate at the country level in every major world region. Furthermore, there is a negative association between women’s employment and unmet need for family planning and a positive association between women’s country-level employment and modern contraception use in all regions. Nonetheless, our results suggest important variation depending on the type of employment. Generally speaking, there is a negative correlation between nonagricultural employment and TFR and unmet need for family planning, and a positive correlation between nonagricultural employment and contraceptive use. On the other hand, there is a positive correlation between agricultural employment and TFR and unmet need for family planning, and a negative correlation between agricultural employment and contraceptive use.

While our main findings are similar cross-regionally, there are a number of important regional differences in the magnitude of these associations. On one hand, the negative associations between women’s employment and TFR and unmet need for family planning are significantly larger for Latin America than any other region, as is the positive association between women’s employment and modern contraceptive use. In part, this could be related to the fact that Latin American countries in our study underwent both a large fertility transition and a dramatic increase in women’s employment during the period of our study. On the other hand, most of the countries in Europe/North America had already undergone the fertility transition by the time period covered in our study, and many already had work–family reconciliation policies that helped ease potential incompatibilities. At the other extreme, many countries in Asia and Africa did not undergo such dramatic transformations, and the fact that a high share of women’s employment continues to be concentrated in agriculture in these regions could help explain why magnitudes of the correlation between employment and fertility/reproductive health outcomes are significantly smaller than in other regions.

Although our study provides an important global overview of employment and fertility, it has a number of limitations. First, our use of aggregate data prevents us from making individual-level inferences about associations between women’s employment and fertility. However, the use of aggregate data also has advantages: the experience of living in a country where many women are employed may have important spillover effects even among unemployed women; these could be captured by our analyses. A second limitation of our analysis is that we cannot address the directionality of the employment and fertility correlation, and in particular whether employment leads to higher fertility or fertility leads to more employment. It is possible (and likely) that both could be true. (The same goes for correlations between employment and modern contraceptive use/unmet need for family planning.) A third limitation of our analysis is that our measure of fertility (TFR) is age standardized but our other measures (such as employment) are not, which implies that changes in a country’s age structure could have some bearing on the empirical associations presented here.

Finally, it is important to note that our results represent associations only; there may be unobserved time-varying factors at the country level that help explain the correlations between employment and fertility/contraceptive use reported in our paper. For example, population age structures could change in ways that are favorable for economic growth and changes in living standards, both of which often correlate with employment and fertility (although since age structure is partly endogenous to TFR, it might be complicated to look at a correlation between employment and TFR net of age structure). At the same time, there could be government or policy changes related to reproduction, contraceptive dissemination, or women’s economic empowerment, all of which would be relevant for the variables of interest in our study. Likewise, over time, patterns of both internal and external migration could change, which would be relevant, since migration is often correlated with both employment and fertility outcomes.

To the best of our knowledge, this paper represents the most complete global exploration of the employment and fertility correlation to date, covering a wide range of countries and data sources. We have widened the employment–fertility debate to include a greater range of reproductive health outcomes as opposed to the narrower focus on fertility that is common in the literature. Our analysis also enhances conversations about the mechanisms through which employment is associated with fertility change by bringing together literature from low- and high-income countries. The dominant approach in the sociological literature on high-income countries attributes the negative correlation between women’s employment and fertility to the logistical incompatibilities women face in combining child care and employment outside the home (Brinton and Lee 2016; McDonald 2000a, 2000b). On the other hand, in low-income countries, wage employment is often conceptualized as empowering by improving women’s ability to bargain over fertility and family decisions (Anderson and Eswaran 2009; Duflo 2012; Narayan-Parker 2005). Bringing these literatures into conversation with each other raises the important possibility that empowerment may help explain some of what we see in high-income countries and that incompatibility may explain some of what we see in low-income countries. Taken together, these approaches provide a more complete and nuanced understanding of the mechanisms between employment and fertility in a truly global context.

## Figures and Tables

**Table 1:** Descriptive statistics

		Women’s employment rate		Total fertility rate	
	N countries	Mean value	Mean # observations (min.–max.)	Mean value	Mean # observations (min.–max)
Total	174	53.2	40.0	3.7	50.9
			(1–59)		(19–56)
1: Europe/North America	42	64.4	47.8	1.7	55.2
			(8–59)		(43–56)
2: Latin America	32	46.7	42.8	3.2	51.5
			(1–59)		(23–56)
3: Africa	48	55.1	33.1	5.7	48.8
			(1–59)		(28–56)
4: Asia	52	46.0	38.8	3.6	49.1
			(4–59)		(19–56)

*Sources*: IPUMS International, ILO, DHS, LIS, UN Population.

*Notes*: See Appendix Table A-1 for the list of countries included in each region.

## Footnotes

3 In the last two decades, the labor force participation of both women and men has decreased (ILO 2016).

4 Informal employment is characterized by jobs that are not covered by labor law or social protection and are often poorly compensated (ILO 2015).

5 We use linear interpolation to fill gaps between observed years of data, and we do not extrapolate outside the range of years included in the data. For instance, if we had data for France between 1975 and 2010 in five-year intervals, the linear interpolation method would only impute values between those five-year intervals, resulting in a yearly series from 1975 to 2010. Thus this method imputes values to complete the time series between the first and the last year of observed data, but it does not generate single-year data between 1960 and 2015 for all countries. This linear interpolation method on average adds only one year of data in the analysis of the association between employment and TFR and about 1.3 years of data in analysis of the association between employment type and TFR. Linear interpolation does not add additional years of data on analyses that look at contraceptive use or unmet need for contraception because these data are already imputed in the original source.

6 In some instances, different methods are used to calculate TFR. To ensure consistency, we select one method per country, preferring the direct method when available. Results (available upon request) are robust due to including only countries that use the direct method.

7 Formally, unmet need for family planning is calculated by summing (a) the number of women of reproductive age (married or in unions) who are not using contraception, are fecund, and desire to either stop childbearing or to postpone their next birth for at least two years; (b) pregnant women whose current pregnancies were unwanted or mistimed; and (c) women in postpartum amenorrhea who are not using contraception and, at the time they became pregnant, had wanted to delay or prevent the pregnancy. This total is divided by the number of women of reproductive age (15–49) who are married or in a union. The result is multiplied by 100.

8 While employment is on the right-hand side in the linear associations in our paper, results are substantively the same if fertility is instead on the right-hand side.

## Appendix

**Table A-1:** List of countries by region and number of observations

1: Europe/North America, NZ, Australia			2: Latin America			3: Africa			4: Asia		
ISO3	Country	#	ISO3	Country	#	ISO3	Country	#	ISO3	Country	#
8: ALB	Albania	12	28: ATG	Antigua and Barbuda	30	12: DZA	Algeria	40	4: AFG	Afghanistan	36
36: AUS	Australia	55	32: ARG	Argentina	56	24: AGO	Angola	29	31: AZE	Azerbaijan	17
40: AUT	Austria	55	44: BHS	Bahamas	25	72: BWA	Botswana	21	48: BHR	Bahrain	38
56: BEL	Belgium	55	52: BRB	Barbados	43	108: BDI	Burundi	36	50: BGD	Bangladesh	42
70: BIH	Bornia	9	68: BOL	Bolivia	40	120: CMR	Cameroon	39	51: ARM	Armenia	19
100: BGR	Bulgaria	51	76: BRA	Brazil	50	132: CPV	Cabo Verde	34	64: BTN	Bhutan	8
112: BLR	Belarus	26	84: BLZ	Belize	21	148: TCD	Chad	14	96: BRN	Brunei	46
124: CAN	Canada	53	152: CHL	Chile	55	174: COM	Comoros	25	104: MMR	Myanmar	32
191: HRV	Croatia	25	170: COL	Colombia	51	178: COG	Congo	27	116: KHM	Cambodia	52
203: CZE	Czechia	25	188: CRI	Costa Rica	43	180: COD	Dem Rep Congo	8	144:LKA	Sri Lanka	48
208: DNK	Denmark	56	192: CUB	Cuba	41	204: BEN	Benin	37	156: CHN	China	29
233: EST	Estonia	27	214: DOM	Dominican Republic	56	231: ETH	Ethiopia	20	158: TWN	Taiwan	38
246: FIN	Finland	56	218: ECU	Ecuador	54	266: GAB	Gabon	18	196: CYP	Cyprus	40
250: FRA	France	54	222: SLV	El Salvador	53	270: GMB	Gambia	29	242: FJI	Fiji	43
276: DEU	Germany	33	254: GUF	French Guiana	30	288: GHA	Ghana	52	258: PYF	French Polynesia	29
300: GRC	Greece	55	312: GLP	Guadeloupe	32	324: GIN	Guinea	20	268: GEO	Georgia	17
348: HUN	Hungary	56	320: GTM	Guatemala	50	384: CIV	Cote d’Ivoire	33	275: PSE	Palestine	15
352: ISL	Iceland	56	328: GUY	Guyana	34	404: KEN	Kenya	7	296: KIR	Kiribati	33
372: IRL	Ireland	50	332: HTI	Haiti	35	426: LSO	Lesotho	15	316: GUM	Guam	21
380: ITA	Italy	55	340: HND	Honduras	40	430: LBR	Liberia	50	344: HKG	Hong Kong	50
428: LVA	Latvia	27	388: JAM	Jamaica	22	434: LBY	Libya	2	356: IND	India	32
440: LTU	Lithuania	27	474: MTQ	Martinique	48	450: MDG	Madagascar	40	360:IDN	Indonesia	44
442: LUX	Luxembourg	56	484: MEX	Mexico	45	454: MWI	Malawi	32	364:IRN	Iran	40
470: MLT	Malta	31	533: ABW	Aruba	21	466: MLI	Mali	39	368: IRQ	Iraq	34
498: MDA	Moldova	26	558: NIC	Nicaragua	39	478: MRT	Mauritania	13	376: ISR	Israel	33
499: MNE	Montenegro	5	591: PAN	Panama	56	480: MUS	Mauritius	33	392: JPN	Japan	56
528: NLD	Netherlands	56	600: PRY	Paraguay	37	504: MAR	Morocco	52	398: KAZ	Kazakhstan	14
554: NZL	New Zealand	30	604: PER	Peru	55	508: MOZ	Mozambique	44	400: JOR	Jordan	54
578: NOR	Norway	55	630: PRI	Puerto Rico	56	516: NAM	Namibia	22	410: KOR	South Korea	56
616: POL	Poland	56	662: LCA	Saint Lucia	13	562: NER	Niger	38	414: KWT	Kuwait	51
620: PRT	Portugal	56	740: SUR	Suriname	50	566: NGA	Nigeria	48	417: KGZ	Kyrgyzstan	27
642: ROU	Romania	50	780: TTO	Trinidad and Tobago	43	638: REU	Reunion	52	418: LAO	Laos	21
643: RUS	Russia	27	858: URY	Uruguay	32	646: RWA	Rwanda	37	422: LBN	Lebanon	4
688: SRB	Serbia	10	862: VEN	Venezuela	52	678: STP	Sao Tome	11	446: MAC	Macao	56
703: SVK	Slovakia	25				686: SEN	Senegal	26	458: MYS	Malaysia	36
705: SVN	Slovenia	25				690: SYC	Seychelles	45	462: MDV	Maldives	38
724: ESP	Spain	46				694: SLE	Sierra Leone	12	496: MNG	Mongolia	13
752: SWE	Sweden	51				710: ZAF	South Africa	54	512: OMN	Oman	16
756: CHE	Switzerland	56				716: ZWE	Zimbabwe	33	524: NPL	Nepal	35
804: UKR	Ukraine	36				729: SDN	Sudan	39	548: VUT	Vanuatu	31
807: MKD	Macedonia	23				748: SWZ	Eswatini	48	586: PAK	Pakistan	40
826: GBR	United Kingdom	43				768: TGO	Togo	32	598: PNG	Papua New Guinea	34
840: USA	United States	56				788: TUN	Tunisia	48	608: PHL	Philippines	53
						800: UGA	Uganda	22	626: TLS	Timor-Leste	10
						818: EGY	Egypt	55	634: QAT	Qatar	30
						834: TZA	Tanzania	37	682: SAU	Saudi Arabia	24
						854: BFA	Burkina Faso	30	702: SGP	Singapore	46
						894: ZMB	Zambia	33	704: VNM	Viet Nam	26
									760: SYR	Syria	45
									762: TJK	Tajikistan	6
									764: THA	Thailand	40
									776: TON	Tonga	29
									784: ARE	Arab Emirates	35
									792: TUR	Turkey	44
									882: WSM	Samoa	52
									887: YEM	Yemen	19

*Note*: The number of observations is the number of years for which both women’s employment and fertility measures are available.

**Table A-2:** Multivariate regression analysis of the association between wage employment and TFR, 1960–2015, including country fixed effects

	Pooled	Pooled	Region 1	Region 1	Region 2	Region 2	Region 3	Region 3	Region 4	Region 4
	All countries	All countries	Europe/North America	Europe/North America	Latin America	Latin America	Africa	Africa	Asia	Asia
Women’s employment rate	−0.0465***	−0.0302***	−0.0159***	−0.0135***	−0.0567***	−0.0548***	−0.0578***	−0.0311***	−0.0531***	−0.0284***
	(0.00119)	(0.00108)	(0.000772)	(0.000824)	(0.00193)	(0.00251)	(0.00293)	(0.00280)	(0.00297)	(0.00243)
GDP		−0.000145***		7.34e-05		0.000450*		−0.00449		−0.000150***
		(3.78e-05)		(0.000283)		(0.000243)		(0.00361)		(4.43e-05)
Gender inequality in secondary education access		−6.458***		−2.431***		−2.209***		−5.701***		−7.609***
		(0.150)		(0.293)		(0.781)		(0.267)		(0.250)
Constant	5.902***	11.09***	2.691***	4.970***	5.745***	7.804***	8.624***	11.95***	5.926***	11.88***
	(0.0638)	(0.132)	(0.0497)	(0.280)	(0.0910)	(0.731)	(0.164)	(0.216)	(0.139)	(0.223)
Country fixed effects										
Observations	5,062	5,062	1,341	1,341	1,007	1,007	1,296	1,296	1,418	1,418
R-squared	0.239	0.448	0.247	0.285	0.471	0.479	0.238	0.445	0.190	0.518
Number of countries	174	174	42	42	32	32	48	48	52	9

*Note*: Standard errors in parentheses.

*** p < 0.01,

** p < 0.05,

* p < 0.1

**Table A-3:** Multivariate regression analysis of the association between women’s employment and modern contraceptive use, 1960–2015, including country fixed effects

	Pooled	Pooled	Region 1	Region 1	Region 2	Region 2	Region 3	Region 3	Region 4	Region 4
	All countries	All countries	Europe/North America	Europe/North America	Latin America	Latin America	Africa	Africa	Asia	Asia
Women’s employment rate	0.615***	0.459***	0.567***	0.543***	0.942***	0.857***	0.430***	0.132***	0.390***	0.152***
	(0.0134)	(0.0125)	(0.0255)	(0.0270)	(0.0197)	(0.0258)	(0.0304)	(0.0268)	(0.0281)	(0.0203)
GDP		0.00371***		0.0256		0.0118***		0.191***		0.00272***
		(0.000433)		(0.0284)		(0.00204)		(0.0355)		(0.000359)
Gender inequality in secondary education access		61.24***		25.67***		19.70**		66.44***		74.11***
		(1.690)		(9.711)		(8.056)		(2.614)		(1.941)
Constant	6.519***	−42.95***	18.53***	−5.914	4.086***	−13.74*	−4.734***	−47.61***	18.73***	−40.01***
	(0.715)	(1.501)	(1.646)	(9.349)	(0.940)	(7.538)	(1.688)	(2.125)	(1.284)	(1.763)
Country fixed effects										
Observations	5,032	5,032	1,300	1,300	1,040	1,040	1,300	1,300	1,392	1,392
R-squared	0.303	0.456	0.282	0.286	0.694	0.704	0.138	0.450	0.126	0.587
Number of countries	168	168	40	40	31	31	47	47	50	50

*Note*: Standard errors in parentheses.

*** p < 0.01,

** p < 0.05,

* p < 0.1

**Table A-4:** Multivariate regression analysis of the association between women’s employment and unmet need for modern family planning, 1960–2015, including country fixed effects

	Pooled	Pooled	Region 1	Region 1	Region 2	Region 2	Region 3	Region 3	Region 4	Region 4
	All countries	All countries	Europe/North America	Europe/North America	Latin America	Latin America	Africa	Africa	Asia	Asia
Women’s employment rate	−0.305***	−0.263***	−0.531***	−0.503***	−0.451***	−0.430***	−0.125***	−0.0187	−0.0851***	−0.0159
	(0.00820)	(0.00851)	(0.0241)	(0.0255)	(0.0109)	(0.0142)	(0.0144)	(0.0141)	(0.0114)	(0.0102)
GDP		−0.00186***		−0.0529**		−0.00653***		−0.106***		−0.00143***
		(0.000293)		(0.0268)		(0.00113)		(0.0186)		(0.000180)
Gender inequality in secondary education access		−15.86***		−27.32***		5.903		−23.44***		−21.16***
		(1.147)		(9.161)		(4.437)		(1.371)		(0.973)
Constant	44.06***	56.92***	58.74***	84.94***	46.71***	40.94***	38.52***	54.53***	33.03***	50.01***
	(0.439)	(1.018)	(1.555)	(8.820)	(0.519)	(4.151)	(0.799)	(1.114)	(0.522)	(0.884)
Country fixed effects										
Observations	5,032	5,032	1,300	1,300	1,040	1,040	1,300	1,300	1,392	1,392
R-squared	0.221	0.256	0.279	0.285	0.630	0.644	0.057	0.263	0.040	0.309
Number of countries	168	168	40	40	31	31	47	47	50	50

*Note*: Standard errors in parentheses.

*** p < 0.01,

** p < 0.05,

* p < 0.1

**Table A-5:** Multivariate regression analysis of the association between women’s employment and TFR by employment type (agricultural versus nonagricultural), 1960–2015, including country fixed effects

	Pooled	Pooled	Region 1	Region 1	Region 2	Region 2	Region 3	Region 3	Region 4	Region 4
	All countries	All countries	Europe/North America	Europe/North America	Latin America	Latin America	Africa	Africa	Asia	Asia
	Agr	non-Agr	Agr	non-Agr	Agr	non-Agr	Agr	non-Agr	Agr	non-Agr
Women’s employment rate	0.0316***	−0.0316***	0.0253***	−0.0253***	0.0584*	−0.0584*	0.0270***	−0.0270***	0.0986***	−0.0986***
	(0.00264)	(0.00264)	(0.00893)	(0.00893)	(0.0329)	(0.0329)	(0.00291)	(0.00291)	(0.0141)	(0.0141)
Constant	2.080***	5.237***	1.678***	4.207***	2.268***	8.104**	2.736***	5.432***	1.792***	11.65***
	(0.0135)	(0.253)	(0.0140)	(0.880)	(0.0888)	(3.207)	(0.0376)	(0.264)	(0.108)	(1.303)
Country fixed-effects										
Observations	1,044	1,044	462	462	242	242	140	140	200	200
R-squared	0.130	0.130	0.018	0.018	0.014	0.014	0.409	0.409	0.219	0.219
Number of countries	85	85	28	28	18	18	15	15	24	24

*Note*: Standard errors in parentheses.

*** p < 0.01,

** p < 0.05,

* p < 0.1

**Table A-6:** Multivariate regression analysis of the association between women’s employment and modern contraceptive use by employment type (agricultural versus nonagricultural), 1960–2015, including country fixed effects

	Pooled	Pooled	Region 1	Region 1	Region 2	Region 2	Region 3	Region 3	Region 4	Region 4
	All countries	All countries	Europe/North America	Europe/North America	Latin America	Latin America	Africa	Africa	Asia	Asia
	Agr	non-Agr	Agr	non-Agr	Agr	non-Agr	Agr	non-Agr	Agr	non-Agr
Women’s employment rate	−0.514***	0.514***	−3.434***	3.434***	0.399	−0.399	−0.459***	0.459***	−0.734***	0.734***
	(0.0364)	(0.0364)	(0.166)	(0.166)	(0.437)	(0.437)	(0.0369)	(0.0369)	(0.134)	(0.134)
Constant	58.05***	6.659*	66.37***	−277.1***	58.55***	98.44**	49.50***	3.600	53.62***	−19.83
	(0.190)	(3.492)	(0.267)	(16.39)	(1.197)	(42.56)	(0.462)	(3.348)	(1.049)	(12.34)
Country fixed-effects										
Observations	1,081	1,081	456	456	255	255	149	149	221	221
R-squared	0.167	0.167	0.498	0.498	0.004	0.004	0.542	0.542	0.133	0.133
Number of countries	85	85	26	26	19	19	17	17	23	23

*Note*: Standard errors in parentheses.

*** p < 0.01,

** p < 0.05,

* p < 0.1

**Table A-7:** Multivariate regression analysis of the association between women’s employment and unmet need for modern family planning by employment type (agricultural versus nonagricultural), 1960–2015, including country fixed effects

	Pooled	Pooled	Region 1	Region 1	Region 2	Region 2	Region 3	Region 3	Region 4	Region 4
	All countries	All countries	Europe/North America	Europe/North America	Latin America	Latin America	Africa	Africa	Asia	Asia
	Agr	non-Agr	Agr	non-Agr	Agr	non-Agr	Agr	non-Agr	Agr	non-Agr
Women’s employment rate	0.265***	−0.265***	3.127***	−3.127***	−0.292	0.292	0.205***	−0.205***	0.498***	−0.498***
	(0.0267)	(0.0267)	(0.161)	(0.161)	(0.291)	(0.291)	(0.0225)	(0.0225)	(0.0799)	(0.0799)
Constant	20.69***	47.24***	14.33***	327.0***	21.34***	−7.819	23.59***	44.13***	22.64***	72.41***
	(0.139)	(2.562)	(0.259)	(15.88)	(0.798)	(28.38)	(0.282)	(2.044)	(0.627)	(7.380)
Country fixed-effects										
Observations	1,081	1,081	456	456	255	255	149	149	221	221
R-squared	0.090	0.090	0.467	0.467	0.004	0.004	0.389	0.389	0.165	0.165
Number of countries	85	85	26	26	19	19	17	17	23	23

*Note*: Standard errors in parentheses.

*** p < 0.01,

** p < 0.05,

* p < 0.1
